# Novel Circoviruses Detected in Feces of Sonoran Felids

**DOI:** 10.3390/v12091027

**Published:** 2020-09-15

**Authors:** Natalie Payne, Simona Kraberger, Rafaela S Fontenele, Kara Schmidlin, Melissa H Bergeman, Ivonne Cassaigne, Melanie Culver, Arvind Varsani, Koenraad Van Doorslaer

**Affiliations:** 1 Genetics Graduate Interdisciplinary Program, University of Arizona, Tucson, AZ 85719, USA; nataliermercer@email.arizona.edu; 2 The Biodesign Center for Fundamental and Applied Microbiomics, Center for Evolution and Medicine, School of Life Sciences, Arizona State University, Tempe, AZ 85287-5001, USA; simona.kraberger@asu.edu (S.K.); rafasfontenele@asu.edu (R.S.F.); kara.schmidlin@asu.edu (K.S.); 3 School of Animal and Comparative Biomedical Sciences, University of Arizona, Tucson, AZ 85721, USA; mhbergem@asu.edu; 4 Primero Conservation, Box 16106, Portal, AZ 85632, USA; icassaigne@yahoo.com; 5 U.S. Geological Survey, Arizona Cooperative Fish and Wildlife Research Unit, University of Arizona, Tucson, AZ 85721, USA; culver@ag.arizona.edu; 6 School of Natural Resources and the Environment, University of Arizona, Tucson, AZ 85721, USA; 7 Structural Biology Research Unit, Department of Integrative Biomedical Sciences, University of Cape Town, Observatory, Cape Town 7701, South Africa; 8 The BIO5 Institute, Department of Immunobiology, Cancer Biology Graduate Interdisciplinary Program, UA Cancer Center, University of Arizona Tucson, Tucson, AZ 85724, USA

**Keywords:** *Circoviridae*, circoviruses, bobcat, *Lynx rufus*, Sonoran Desert, Sonoran felid associated (Sonfela) circoviruses

## Abstract

Sonoran felids are threatened by drought and habitat fragmentation. Vector range expansion and anthropogenic factors such as habitat encroachment and climate change are altering viral evolutionary dynamics and exposure. However, little is known about the diversity of viruses present in these populations. Small felid populations with lower genetic diversity are likely to be most threatened with extinction by emerging diseases, as with other selective pressures, due to having less adaptive potential. We used a metagenomic approach to identify novel circoviruses, which may have a negative impact on the population viability, from confirmed bobcat (*Lynx rufus*) and puma (*Puma concolor*) scats collected in Sonora, Mexico. Given some circoviruses are known to cause disease in their hosts, such as porcine and avian circoviruses, we took a non-invasive approach using scat to identify circoviruses in free-roaming bobcats and puma. Three circovirus genomes were determined, and, based on the current species demarcation, they represent two novel species. Phylogenetic analyses reveal that one circovirus species is more closely related to rodent associated circoviruses and the other to bat associated circoviruses, sharing highest genome-wide pairwise identity of approximately 70% and 63%, respectively. At this time, it is unknown whether these scat-derived circoviruses infect felids, their prey, or another organism that might have had contact with the scat in the environment. Further studies should be conducted to elucidate the host of these viruses and assess health impacts in felids.

## 1. Introduction

The Sonoran Desert is a unique ecosystem in which four species of felids are known to coexist: pumas (*Puma concolor*), bobcats (*Lynx rufus*), ocelots (*Leopardus pardalis*), and jaguars (*Panthera onca*) [1]. These felids play a crucial role in maintaining a functional ecosystem. Pumas mainly regulate populations of ungulates, including deer, bighorn sheep, and javelina [2,3,4], while bobcats and ocelots tend to prey upon small mammals, such as lagomorphs and rodents, and reptiles [3,5,6,7]. Ocelots and jaguars are recognized as endangered in the region [8,9,10]; however, the status of all four felid species are likely threatened by shared environmental pressures, including drought [11], habitat fragmentation and encroachment (which can lead to human-wildlife conflict), and emerging diseases. While antibodies to canine distemper virus (CDV) have been detected in Sonoran jaguars [12] and antibodies to CDV, feline panleukopenia virus, feline calicivirus, and feline enteric coronavirus have been detected in pumas from southern Arizona [13], other viruses circulating in populations of Sonoran felids are largely unknown. Cataloging the diversity of viruses present in these felids could reveal an abundance of both known and novel viruses; although most viruses are not pathogenic, some may cause disease and be relevant to conservation.

High throughput sequencing technologies have allowed for unprecedented advances in identifying known and novel viruses and characterizing viral communities through viral metagenomics. Taking advantage of metagenomic approaches to monitor viral communities associated with wildlife could be instrumental for conservation; however, this is not routinely performed. Altered viral evolutionary dynamics (largely due to anthropogenic factors such as facilitating viral movement around the world, spillover from domestic animals, increasingly dense populations of wildlife due to habitat encroachment, and climate change) and altered exposure of wildlife to viruses through vector range expansion create conditions for accelerated emergence of viruses, some of which may cause new disease outbreaks in wildlife populations [14,15]. Notable examples include the spillover of feline leukemia virus (FeLV) from domestic cats into the endangered Florida panther [16] and spillover of CDV from domestic dogs into wildlife populations within Serengeti National Park, Tanzania, affecting spotted hyenas, African lions, and other species [17,18]. This may be especially problematic for already threatened populations, as small populations typically have lower genetic diversity (and possibly stress-induced immunosuppression) and, therefore, decreased adaptive potential to assist survival of a proportion of the population experiencing the effects of a novel viral disease [15,19,20,21].

Genomes from several families of circular Rep-encoding single-stranded DNA viruses (CRESS-DNA viruses) are part of the phylum *Cressdnaviricota* [22] and have been identified in fecal samples of other mammals, including domestic cats [23,24], bobcats, African lions [25], capybaras [26], and Tasmanian devils [27]. *Circoviridae* is one of the families in the *Cressdnaviricota* phylum and is composed of the genera *Circovirus* and *Cyclovirus*. Circoviruses have ambisense genomes of approximately 1.7–2.1 kb in length and encode two proteins, Rep and the capsid protein (CP) [28]. Circoviruses have implications for wildlife management because they are associated with disease in some vertebrates, including life-threatening hemorrhagic gastroenteritis in dogs [29,30,31], psittacine beak and feather disease in parrots [32], and postweaning multisystemic wasting syndrome in pigs [33,34]. Importantly, several studies suggest that these life-threatening diseases may be largely due to coinfection with porcine parvovirus or porcine reproductive and respiratory syndrome virus [35,36], or canine coronavirus, canine parvovirus, or CDV [37,38,39], in pigs and dogs respectively.

No circoviruses are known to infect felids, although a cyclovirus (feline associated cyclovirus 1) has been identified in the feces of domestic cats [23]. Additionally, a feline stool-associated circular DNA CRESS-DNA virus has recently been identified from cats with diarrhea [24]. Endogenous fragments of circoviruses have also been detected in feline genomes, indicating the susceptibility of the ancestors of modern felids to circovirus infection [40,41].

Here we used a metagenomic approach to identify novel circoviruses in the feces of two species of Sonoran felids, the puma and bobcat; although not endangered, knowledge of viral threats facing these species could help prevent future population decline, as well as indicate potential threats to the endangered ocelot and jaguar. For the two novel circoviruses identified, we sought to determine relationships with known circoviruses and characterize their genomes. These novel feline feces associated circoviruses may represent the first known feline circoviruses.

## 2. Material and Methods

### 2.1. Sample Collection and Source Identification

Scat samples from bobcats and pumas were collected from Sonora, Mexico, between 2012 and 2014. The samples were desiccated at room temperature prior to shipping and long-term storage at −20 °C. To determine the species, DNA was extracted by swabbing the scat surface. The swab was deposited into lysis buffer and DNA extracted using Qiagen’s DNeasy Blood and Tissue Kit as previously described by Cassaigne et al. [4]. This DNA was used as template for PCR of the mitochondrial cytochrome B gene [42] with confirmation by Sanger sequencing of the amplicon (~470 bp region) as previously described [43].

### 2.2. Fecal Viral Metagenomics

We randomly selected fecal samples (bobcats (*n* = 9) and pumas (*n* = 13)) for this study. Of each of the fecal samples, 5 g was homogenized in SM buffer and the homogenate was centrifuged at 6000× *g* for 10 min. The supernatant was sequentially filtered through 0.45 μm and 0.2 μm syringe filters and viral particles in the filtrate were precipitated with 15% (*w/v*) PEG-8000 with overnight incubation at 4 °C followed by centrifugation at 10,000 ×*g* as described in Fontenele et al. [26]. The pellet was resuspended in 500μL of SM Buffer and 200μL of this was used for viral DNA extraction using the High Pure Viral Nucleic Acid Kit (Roche Diagnostics, Indianapolis, IN, USA). Circular viral DNA was amplified by rolling circle amplification (RCA) using the Illustra TempliPhi Amplification Kit (GE Healthcare, Chicago, IL, USA). Sequencing libraries were prepared from the RCA products using the Nextera DNA Flex Library Prep Kit (Illumina, San Diego, CA, USA) and sequenced on an Illumina HiSeq 4000 (2 × 100 bp). The paired-end raw reads were trimmed using default settings within Trimmomatic v0.39 [44] and the trimmed reads were de novo assembled using k-mer values of 33, 66, and 77 within metaSPAdes v 3.12.0 [45]. Contigs greater than 500 nucleotides were analyzed by BLASTx [46] against a local viral protein database constructed from available NCBI RefSeq viral protein sequences (https://ftp.ncbi.nlm.nih.gov/refseq/release/viral/).

### 2.3. Recovery of Circovirus Genomes

Based on the de novo assembled contigs (>750 nts) that had BLASTx hits to circovirus sequences, two pairs of abutting primers were designed manually to recover and verify the full genomes of circoviruses: UoA14_16F 5′-CTATAGAACAGATATGCAAATTATGGCCGG-3′ and UoA14_16R 5′-ATATCTCAAAAAGAGGAACCGAAACCTTGG-3′ (complementarity to *cp* gene/stem loop region) and UoA15F 5′-GACCGATACCCATTGAAAGTGGAGACTAAG-3′ and UoA15R 5′-CATCACTCGAAGCAGGTCATCATAG-3′ (complementary to the *rep* gene region). As a template, 0.5 μL RCA product was used with KAPA HiFi HotStart DNA Polymerase (Kapa Biosystems, Wilmington, MA, USA) and the specific abutting primers described above were used for each of the fecal samples to screen and recover the full genomes of the circoviruses using the manufacturer’s recommended thermal cycling conditions.

The PCR amplicons were resolved on a 0.7% agarose gel, recovered with gel purification, cloned into the plasmid pJET1.2 (ThermoFisher, Waltham, MA, USA), and Sanger-sequenced at Macrogen Inc. (Seoul, South Korea) by primer walking. The Sanger sequence contigs were assembled using the “assembly module” in Geneious Prime v1 [47].

### 2.4. Sequence Analyses

Open reading frames in the genomes were identified using ORFfinder (https://www.ncbi.nlm.nih.gov/orffinder/). The genomes and amino acid sequences of Rep and CP of representative circoviruses and those identified in this study were aligned using MUSCLE [48], and pairwise percent identities were obtained using SDT v1.2 [49] (File S1). The optimal substitution model based on Akaike information criterion with correction for small sample size (AICc) for the genome alignment was identified as GTR+I+G using jModelTest 2 [50,51], and ProtTest 3 [52] identified LG+I+G as the optimal model for the Rep alignment and VT+I+G+F as the optimal model for the CP alignment. Phylogenetic analyses for each alignment were performed with PhyML 3.0 [53]. For visualization purposes, all trees were rooted with sequences from the duck associated cyclovirus 1 (GenBank: KY851116) and horse associated cyclovirus 1 (GenBank: KR902499) (not shown in the tree). Branches with SH-like aLRT support less than 0.8 [53,54] were collapsed using ips [55] and ape [56] packages in R [57]. The viral genomes described in this manuscript were submitted to GenBank (accession numbers: MT610105–MT610107).

## 3. Results and Discussion

**Figure 1:**
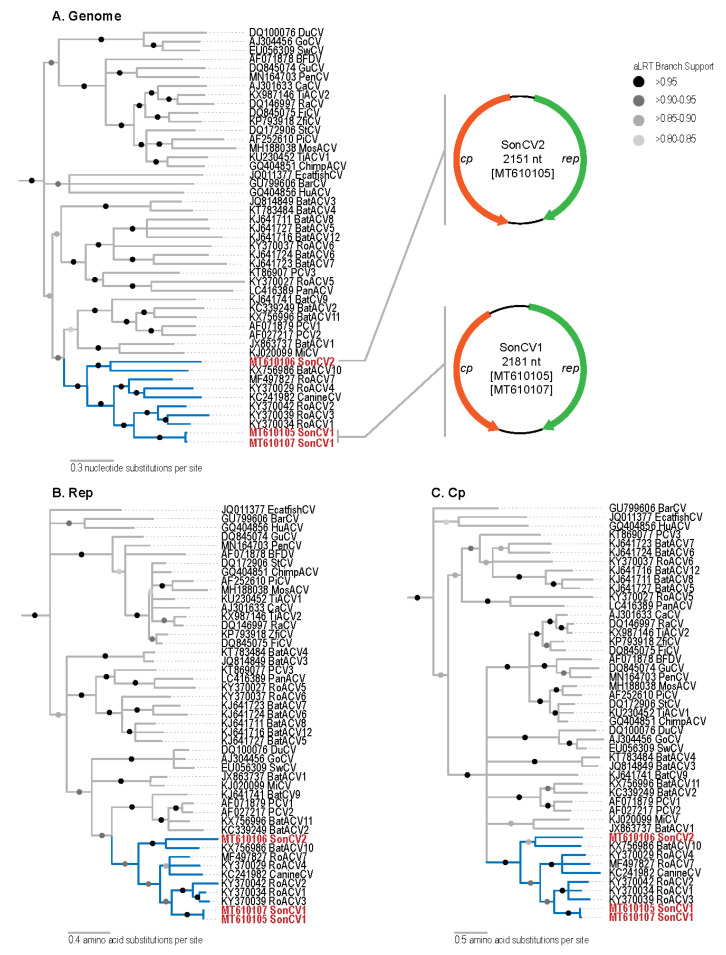
(**A**) Maximum likelihood phylogenetic tree of genome sequences of three Sonoran felid associated (Sonfela) circovirus (SonCV) genomes (red font with clade highlighted in blue) and other representative circoviruses and genome organizations of the two novel SonCVs. (**B**) Maximum likelihood tree of Rep amino acid sequences of the circoviruses including those of SonCVs. (**C**) Maximum likelihood tree of CP amino acid sequences of the circoviruses including those of SonCVs.

Based on the metagenomic analysis, we assembled a partial viral genome in two of the samples. Based on this partial sequence data, we designed abutting primers to screen all the available scat samples. Of the 22 samples screened with the two primer pairs, three circovirus genomes were identified and recovered (Figure 1A) from three fecal samples of bobcats. Two of the genomes (GenBank: MT610105 and MT610107) share greater than 97% pairwise identity to each other (File S1) and are 2181 nucleotides in length, having a Rep coding sequence (CDS) of 906 nucleotides (302 amino acids) on the virion-sense strand and CP CDS of 816 nucleotides (272 amino acids) on the complementary strand. Based on the species-demarcation threshold for circoviruses which is 80% genome-wide identity [28], both of these belong to a new species which we refer to as Sonfela (derived from Sonoran felid associated) circovirus 1. The third genome (GenBank: MT610106) of 2151 nucleotides, referred to as Sonfela circovirus 2, is more distantly related, sharing approximately 61% identity with the two Sonfela circovirus 1 genomes (File S1), and contains a Rep CDS of 864 nucleotides (288 amino acids) on the virion-sense strand and CP CDS of 975 nucleotides (325 amino acids) on the complementary strand. The stem loop and nonanucleotide motif “TAGTATTAC” were identified in the genomes and correspond to the origin of replication. Conserved motifs within Rep (RC endonuclease Motifs I, II, and III and SF3 helicase domains Walker A, Walker B, Motif C, and Arg finger) [58] were all detected.

The genome (Figure 1A) and protein ML phylogenetic trees (Figure 1B,C) demonstrate that canine circovirus (GenBank: KC241982), rodent associated circoviruses (RoACV 1,2,3,4, and 7) (GenBank: KY370034, KY370042, KY370039, KY370029, and MF497827), bat associated circovirus 10 (GenBank: KX756986), and the Sonfela circoviruses cluster in a separate clade with SH-like aLRT support between 0.902–0.997. Sonfela circovirus 1 is most closely related to a group of three rodent-derived viruses (RoACV1–3; GenBank: KY370034, KY370042, and KY370039), sharing a maximum of approximately 70% genome-wide identity, 70% Rep identity, and 60% CP identity with RoACV2 (GenBank: KY370042) (File S1). The phylogenetic trees reveal Sonfela circovirus 2 and bat associated circovirus 10 (GenBank: KX756986) to be sister taxa, sharing approximately 63% genome-wide identity, 64% Rep identity, and 45% CP identity according to SDT; however, pairwise percent identity calculations reveal maximum genome-wide identity with BatACV7 (GenBank: KJ641723) (63.5%) and CP identity with RoACV1 (GenBank: KY370034) (46%) (File S1). Sharing less than 80% genome-wide identity with known circoviruses, both Sonfela circoviruses 1 and 2 represent novel species (File S1).

## 4. Conclusions

Based on the circovirus species demarcation threshold of 80% identity [28], the circovirus genomes identified and recovered in this study represent two new species. These feline associated viral genomes have a typical circovirus length, contain both circovirus Rep and CP CDS (in appropriate orientation), and have a well-defined nonanucleotide sequence.

The health implications of these circoviruses for these populations are currently unclear given the viruses’ true hosts and pathogenicity are unknown. As the viral genomes were derived from scat samples, the circoviruses could have infected the bobcat prey species or the felids themselves or be environmentally derived. The phylogenetic clustering of Sonfela circovirus 1 and several rodent circoviruses suggests the virus may be rodent-derived; similarly, Sonfela circovirus 2 may be bat-derived.

As with these novel feline associated viruses, many of the recently described viruses have not been associated with their mammalian hosts. The lack of formal host association limits our ability to directly interpret the biological relevance of these viruses. However, in the meantime, it is critical to continue to describe the viral diversity associated with unconventional hosts.

To our knowledge, the circoviruses described here may represent the first known feline associated circoviruses. Detection, or lack thereof, of the circoviruses in other tissues within felids could help discern the viruses’ true hosts. Screening for the viruses in sympatric populations of rodents, bats, and other prey species could also be utilized to rule out or confirm the sources of these viruses. If felids are the host for these viruses, affected individuals should be monitored for possible symptoms of disease; however, further investigations linking these viruses to their natural host are needed as well as investigations into the prevalence of the viruses within felid populations in the Sonoran Desert and across the Americas.

## Supplementary Materials

The following are available online at https://www.mdpi.com/1999-4915/12/9/1027/s1, File S1: Pairwise identity matrices of the genome and Rep and CP amino acid sequences of circoviruses.
